# Construction and Application of Text Entity Relation Joint Extraction Model Based on Multi-Head Attention Neural Network

**DOI:** 10.1155/2022/1530295

**Published:** 2022-05-24

**Authors:** Yafei Xue, Jing Zhu, Jing Lyu

**Affiliations:** ^1^School of Computer and Information, Hohai University, Nanjing, Jiangsu 211100, China; ^2^Department of Information Science and Technology, Nanjing Normal University Zhongbei College, Nanjing, Jiangsu 210046, China; ^3^College of Computer and Information Engineering, Xinjiang Agricultural University, Wulumuqi, Xinjiang 830052, China

## Abstract

Entity relationship extraction is one of the key areas of information extraction and is an important research content in the field of natural language processing. Based on past research, this paper proposes a combined extraction model based on a multi-headed attention neural network. Based on the BERT training model architecture, this paper extracts textual entities and relations tasks. At the same time, it integrates the naming entity feature, the terminology labeling characteristics, and the training relationship. The multi-attention mechanism and improved neural structures are added to the model to enhance the characteristic extraction capacity of the model. By studying the parameters of the multi-head attention mechanism, it is shown that the optimal parameters of the multi-head attention are *h* = 8, *dv* = 16, and the classification effect of the model is the best at this time. After experimental analysis, comparing the traditional text entity relationship extraction model and the multi-head attention neural network joint extraction model, the model entity relationship extraction effect was evaluated from the aspects of comprehensive evaluation index *F*1, accuracy rate *P*, and system time consumed. Experiments show: First, in the accuracy indicator, Xception performance is best, reaching 87.7%, indicating that the model extraction feature effect is enhanced. Second, with the increase of the number of iterative times, the verification set curve and the training set curve have increased to 96% and 98%, respectively, and the model has a strong generalization ability. Third, the model completes the extraction of all data in the test set in 1005 ms, which is an acceptable speed. Therefore, the model test results in this article are good, with a strong practical value.

## 1. Introduction

With the increasing progress of Internet technology, information presents a trend of massive growth. In this “big data era,” information data are circulated or used in different forms by means of different vectors. According to Intel's forecast, the global data volume will reach 46ZB in 2022, and the data are increasing at an unimaginable rate every day. More than 90% of the data formats are unstructured data, such as text, website, video, audio, image, etc., and the data in the form of text occupies a large proportion of unstructured data [1]. How to transform the nonstructured information in the text to facilitate structured information for computer understanding has become a critical issue in the field of text mining. Information extraction techniques are therefore produced.

Information extraction is the study of how to extract specified information from massive unstructured natural language texts, and turn unstructured text into structured data, which is convenient for computer storage and understanding [2]. According to the segmentation of automatic content extraction, the research content of information extraction has four aspects: nomenclature identification, referential resolution, entity relationship extraction, and event extraction [3]. Among them, entity relationship extraction is a key ring of information extraction. The traditional approach to information extraction is to treat entity and relation extraction as two completely separate tasks. Due to the loss of correlation information between entities and relationships, the model is prone to erroneous matching problems [4]. Therefore, many scholars have begun to try the joint extraction of entity relationships. The conventional joint extraction model has improved the problem of error propagation to some extent but is limited to the fixed representation of the traditional word vector, and the restriction of the extraction capability of the feature extractor, therefore, encountered in the improvement of the overall effect of the model. It is difficult to break through the bottleneck.

The multi-head self-attention mechanism can capture long sequence information well, and has the characteristics of fast speed and easy interpretation. It has been widely used in natural language processing and other fields [5]. In order to improve the classification performance of the model, this paper combines multiple attention mechanisms and neural network technologies on the basis of past research, and proposes a combination model based on a multi-point attention neural network. It directly extracts the relationship between “entity-relationship-entity” in the text, adding multiple attention mechanisms to the input text from the text feature extraction, encoding the input text from different directions, and enhancing the coding effect of the model. It uses the constructed hierarchical neural network model to integrate the BERT training model with named entity features and part-of-speech tagging features to complete entity feature extraction, subject-object recognition, and entity relationship joint extraction. In this paper, the labeling strategy is used in the sequence labeling model, which breaks the fixed representation method of the traditional word vector and finally improves the performance of the model in this paper.

## 2. Related Discussion

Entity relationship extraction is the semantic relationship between the given entity pair [6]. Text entity relationship extraction is an important part of information extraction, the identification of the most basic task naming entity. As for text information, two important tasks are extracted, namely, entity identification and physical relationships have made great progress. At present, most scholars are based on the pipeline model for named entity recognition and physical relation extraction. It looks into two independent subtasks, respectively, namely, entity identification and physical relationship extraction, and processes both tasks [7]. However, the conduit-based study does not consider the correlation between the two subtasks and the information fusion between the tasks, and can easily lead to problems such as erroneous propagation [8].

Regarding the joint extraction of naming entities and entity relations, the current research results are less, and more steps need to be studied. Based on the relationship extraction method of the feature vector, first build a feature vector for the original text, and then find the relationship between the entities through the statistical learning model [9]. Qiao proposed a relationship extraction method based on the maximum entropy model, which is integrated with the lexical, syntax, and semantic feature vectors [10]. Pagan added the word block information and Word-net information to the built-in feature vector and enhanced the text's syntax information and semantic information [11]. This type of method model is fast, but it is necessary for human design, and the applicability and development prospects have been significantly limited. Fei and Ren proposed in 2014 to use structural prediction methods to combine and extract, better results on the ACE data set [12]. Chen proposed an end-to-end model in 2016, using BILSTM and Tree-LSAT model. They first extracted the input text through BILSTM, the encoding layer was used as a shared layer, and the shortest content tree network extracted physical relations and achieved good results [13]. Cheng and Song first proposed a combinatorial extraction method based on ensemble neural networks in 2017. They used BILSTM network encoding, then utilized LSTM network decoding to generate entities, classify, and extract CNN layers, and extracted entities and relations simultaneously [14]. Korzybski proposed in 2017 the joint extraction method based on the new labeling policy, converts the joint extraction task into a sequence annotation problem, direct end-to-end extraction, the experimental results indicate that the combined extraction results are better than the extraction method based on pipeline mode [15]. Li proposed a joint extraction method based on state transfer in 2018, which transformed the joint extraction task into a state transfer process. First, the input text was encoded and represented by Bi-LSAT, and then the encoding result was used as the input model. The state predicts the next transition action until reaching the final state, and jointly extracts entities and inter-entity relationships [16].

In 2019, Abboudi et al. used a bidirectional long short-term memory network collective attention (Attention) mechanism for relation classification tasks [17]. The Attention layer weights the output of each node of the LSTM instead of just taking the value of the last node. Using the attention mechanism can reduce the influence of noise words in sentences and enhance the influence of keywords. BI-LSAT-Attention is a commonly used model in natural language processing tasks, and it has also achieved good results in relational classification tasks. Wang proposed a model of a convolution neural network combined with a two-layer attention mechanism, using a relatively complex two-layer attention mechanism to highlight as much as possible the part of the sentence that has a greater contribution to the relationship category. From the current research progress, the input layer of most models contains word vector and position vector information. The attention mechanism plays an obvious role. Adding an attention mechanism to the input layer can give weights to each word. This weight can reflect the correlation between the word and the relationship category, and can also realize the correlation between the word and the entity. Recurrent neural network or long short-term memory network plus attention mechanism on the output of all nodes can also reflect the correlation between each word and the type of relationship.

For the named entity recognition task and the relationship classification task, there are mainly two types of methods. One is to use the serial method for extraction. For the input sentence, the named entity recognition task is firstly performed, and then the recognized entities are combined to form entity pairs, the relationship of each entity pair is classified, and finally the entity pairs and relations with entity relations are output as triples. There are three disadvantages of the serial method. First, since the entity recognition task is before the relationship classification task, the output of the named entity recognition is the input of the relationship classification, so the error of the entity recognition module will have a bad impact on the performance of the later relationship classification. It is the problem of error propagation; secondly, the serial method ignores the relationship between the two subtasks, namely, entity recognition and relationship classification. If there is a “Country-President” relationship between “United States” and “Trump,” then the former entity must be of type “Location” and the latter entity of type “Person,” and the serial method cannot use this useful information. Finally, the serial method generates a lot of redundant information. Since the identified entities need to be formed into entity pairs, and then the relationship is classified, those entity pairs that have no relationship will bring a lot of redundant information, which also directly leads to an increase in the error rate.

The ideal joint learning model of named entity recognition and relation extraction should directly output the entity triples with relation through the joint model of entity recognition and relation extraction for the input sentence. This joint model can overcome the shortcomings of the above serial method, and it follows that the model may have a more complex network structure, more parameters to be trained, and a longer training time. The joint learning method based on neural network is an important research content of joint learning tasks at present. The current work is mainly divided into two categories, one is the joint extraction method of entity relations based on parameter sharing, and the other is based on the tagging strategy entity-relationship joint extraction. Among them, the joint extraction method based on parameter sharing is simple and easy to implement, and has extensive and effective applications in many multi-task learning. The new labeling strategy provides a new idea of joint learning, which truly realizes the combination of two subtasks into a sequence labeling problem. Based on this, the joint extraction in this paper will adopt a method based on the labeling strategy, and add a reinforcement learning framework to this labeling strategy to make more improvements and developments, so as to further improve the end-to-end entity-relationship joint extraction task.

Many experts and scholars use traditional neural network, BP neural network, and convolution neural network for the joint extraction model of text entity relationship, but most joint named entity recognition processes is limited by the capabilities of feature extractors. Therefore, in the field of textual research, this paper proposes a combination model based on a text entity relationship based on a multi-head focus neural network. During the relational extraction, the learning ability of the order characteristics between tokens will be enhanced, and the effect of this model is further improved.

## 3. Entity Relationship Joint Extraction Model Construction

Entity relationship combined with extraction requires a collection of entities contained in the original text, and then identifying the relationship between each pair of entities. It is uniformly divided into two tasks, extracts the structured relationship of “entity-relations-entity” three-tuple information from text.

### 3.1. Model Overall Structure

**Figure 1:**
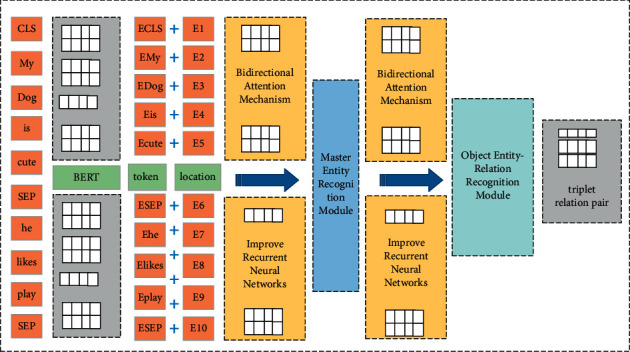
Model overall structure diagram.

This paper proposes a text entity relationship of a text entity based on a multi-head focused neural network. When resolving the relational extraction task, a three-layer linear architecture model is defined, including the text feature extraction layer, the main real identification module, and the guest entity-relationship Identify module. The overall structure diagram is shown in Figure 1.

As can be seen from Figure 1, the underlying is a natural language text feature extraction layer, which uses the two-way encoder representation model that rejudges on Baidu Encyclopedia. The model uses two phased models: the first is the training of the language model, and the second phase uses fine-tuning to solve the downstream task. The model input consists of three parts: the given sequence, the sequence position embedding, the clause embedding and the token embedding ensemble [18]. This model is further fused from the extracted nomenclature characteristics and the labeling feature. Through this processing, the training model of this paper can effectively capture implicit entities and relationship information contained in more text, and more effectively adapt to the relationship extraction task to obtain an easier-to-interpret entity relationship [19]. The intermediate layer is the main entity identification module, which is mainly used for the identification of the main body. The upper layer is a guest entity-relational identification module. This paper adds multiple attention mechanisms and improved neural network models on both modules to further enhance the extraction capabilities of sequential features between TOKENs.

### 3.2. Model Feature Enhancement Processing

Because the previous model is affected by the feature extractor, the feature extraction is limited, resulting in a low accuracy rate of the model for text recognition, so it is necessary to further enhance the feature processing capability of the model. In order to better extract text-related entities, the model is further refined according to task requirements and further document features are extracted, as well as named entity features, mean annotation features, and popularity in documents. The well-trained BERT model is combined to enable this model to effectively adapt to new relationships to extract tasks, and have better interpretation, and more accurate relationships.

**Figure 2:**
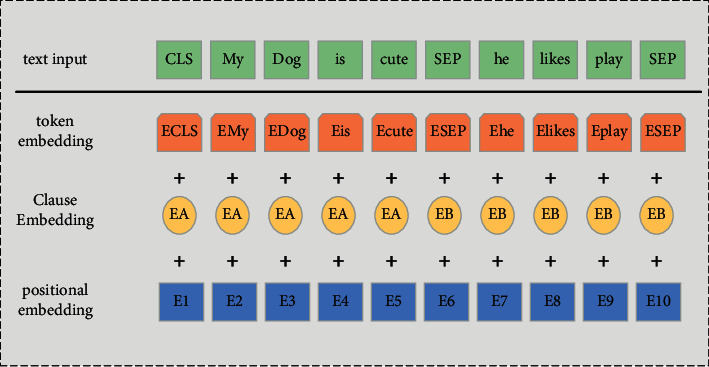
Neural network structure.

When the problem is input to the document into the adjusted neural network language model, the characteristic enhancement representation of each word in the document is obtained, namely, entity feature *E*_NER_, and the morphia label feature *E*_NER_, model processing are calculated as follows:

(1) $$\begin{array}{c} E_{\mathrm{mod}\text{el}}=\text{BERT}\left|Q\right|,\\ E_{\text{f}}=M_{\text{q}}E_{POS}\pm M_{n}E_{NER},\\ S=\tanh\left\{E_{\mathrm{mod}\text{el}}\pm E_{\text{f}}\right\}, \end{array}$$

where BERT is the trained model, *M*_*Q*_, *M*_*n*_ is its parameter matrix, and TANH is a dual-tone orthogonal excitation function equation. The specific neural network structure is shown in Figure 2.

### 3.3. Main Real Body Recognition Module

After the previous section, the extraction of the BERT word in this article and the nomenclature entity, the allomorph feature is fused, the subsection is based on the nerve network language model of the feature enhancement process, and the downstream primary entity identification task is modeled. Since the language model of the Bert structure is composed of an Encoder part in multi-layer transformer, this structure does not learn continuous features, that is, continuous correlation between Token and Token does not fully learn [20]. To this end, an improved circular neural network is added to the model of this article, which improves the gradient explosion of traditional circulating neural network models during training, and greatly enhances the training efficiency of circulating neural network models. The formula is as follows:

(2) $$\begin{array}{c} \tilde{S}=Ci-\text{TELW}\left(S\right), \end{array}$$

where TELW is the decoder function and *Ci* is the memory of the current TELW unit.

**Figure 3:**
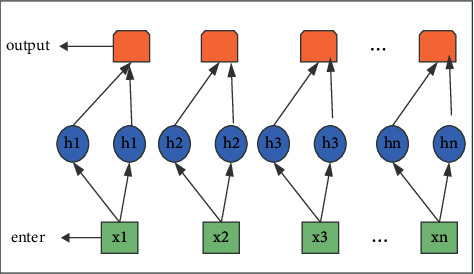
Two-layer perceptual input and output procedure.

Finally, this paper is shown in Figure 3. The two-layer perceptron can learn a lot of continuous features, which improves the efficiency of recognition training. The starting flag bit and the end flag of the final main entity are obtained by two multi-layer perception (MLP).

(3) $$\begin{array}{c} Q_{k}^{\text{start}\cdot R}=\theta \left(M_{\text{start}}y_{k}+d_{\text{start}}\right),\\ Q_{k}^{\text{end}\cdot R}=\theta \left(M_{\text{end}}y_{k}+d_{\text{end}}\right), \end{array}$$

where *Q*^START^_ and *Q*^END^ represent the input sequence, the *k* position is the input, as the main body start tag and end tag.

**Figure 4:**
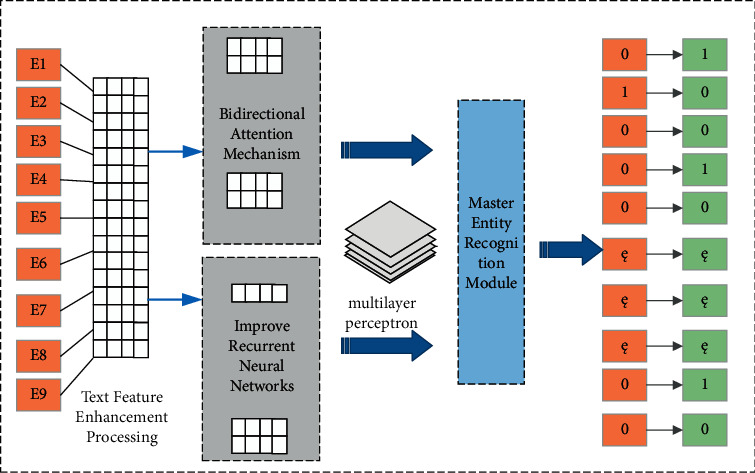
The overall structure of the main real body recognition module.

The overall structure of the main real body recognition module is shown in Figure 4. When text passes through a feature-augmented neural network language model, it learns weak problems by compensating for the sequential features of tokens through recurrent neuron memory network units' loops. Finally, the start flag bit and the end flag of the final main entity are obtained by two-layer multi-layer perception (MLP).

The main real body recognition module identifies the range of the given main body S by optimizing the following functions:

(4) $$\begin{array}{c} Q_{\partial }\left(S_{X}\right)=\overset{L}{\underset{\text{k}=1}{\cap }}\left(Q_{k}^{t}\right)\times l\left(y_{k}^{2}=1\right)\left(1+Q_{k}^{t}\right)\times l\left(y_{k}^{2}=0\right). \end{array}$$

The parameter *L* represents the length of the sentence, *Y*_*K*_ indicates that the value of the *K* position token predicted is 1, and the *Q*_*K*_ represents the value of the TOKEN predicted in the *K* position is 0. When there are multiple primary entities in the sentence, the model is extracted according to the proximity of the main entity. After the start flag *Q*^START^, the nearest end flag bit of the start flag bit is defined as the scope of the subject.

### 3.4. Object Entity-Relationship Identification Module

**Figure 5:**
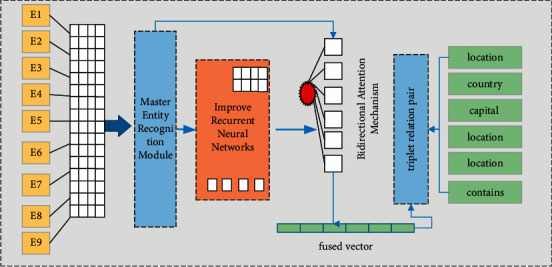
The object entity relationship identification module.

This article constructs a corresponding object entity-relational identification module for the objective entity extraction and relationship mission. The overall structure of the object entity relationship identification module is shown in Figure 5.

As shown, the text establishes a neural network language model through feature enhancement processing. First, the main entity encoding vector *S*_*t*_ is obtained by improving the rear circulating neural network.

(5) $$\begin{array}{c} {\tilde{S}}_{t}=Ci-\text{TELW}\left(S_{t\cdot e}\right). \end{array}$$

This article will incorporate the feature vectors processed by the text feature layer to fuse the resulting vector and main body encoding vector. The fusion vector is further enhanced by the improved circulating neural network unit to compensate for the order of the order characteristics between TOKENs. Finally, the start flag bits of the passenger entity and the end flag bit are obtained by two multi-layer perception (MLP) to obtain two multi-layer perception (MLP).

The multi-head attention mechanism *h* represents the number of attention heads, that is, the number of operations of attention. The more attention heads, the more operations. The number of operations will increase the time consumption of the model, and only the appropriate number of attention heads can improve the performance of the model.

(6) $$\begin{array}{c} \text{Multi Head}\left(G,J,U\right)=\text{Concat}\left[{\text{head}}_{1}+{\text{head}}_{2}+\cdots {\text{head}}_{n}\right]V_{N},\\ {\text{head}}_{n}=\text{Attention}\left[GV^{G},JV^{J},UV^{U}\right]V. \end{array}$$

The multi-head self-attention mechanism network is used to perform further information filtering on the feature input *B*. Through multi-head self-attention, the object entity-relation recognition module can learn more fine-grained text interaction features.

(7) $$\begin{array}{c} B_{T}=S\text{elf}+\text{Attention}\left(S\right). \end{array}$$

The obtained finer granularity hidden layer vector BT is spliced after the encoding vector ST is obtained by obtaining the main substance, the vector *Z*:

(8) $$\begin{array}{c} Z=\left(B_{T}+S_{T}\right). \end{array}$$

Finally, the model will be given the hidden vector *G* to the improved circulatory neural network unit to make up for the learning weakness of the order between TOKENs.

## 4. Experimental Design

### 4.1. Lab Environment

The environment and programs used in this experiment, and the server configuration used in the experiment are shown in Table 1.

The experimental environment of this article is: development language: Python 3.6.0, Snyder in the programming tool Anaconda integrated development environment, and the python third-party tool-kits called gen-sim, learn, Eras, Tensor-flow, etc. Among them, the word segmentation toolbox adopts the Jibba word segmentation package; the splitting of the data set adopts Stratification in the learn toolbox; for the text feature representation method, the traditional text representation method uses TFIDF, LDA, and LSA in the gen-sim toolbox, and the word The vector distributed representation calls Word2vec in the gen sim toolbox; the traditional machine learning classifier model calls the model in the learn toolbox; the deep learning framework uses Eras and Tensor flow; the visualization of the experimental results uses the diplomatist drawing tool.

### 4.2. Experimental Data Set

To evaluate the performance of our method, we use data generated by a multi-head attention neural network, which contains 395 sentences with a total of 3880 test entity-relation pairs. The experimental results in this article will also be compared with the results of other methods. The training data contains 235,982 corpora with a total of 353,000 triples. A large number of data sets contain more than 99% of the text information. In order to further reduce the error and ensure the accuracy of the experimental results, repeated comparison training is carried out during the experiment.

### 4.3. Evaluation Metrics

The evaluation indicators used in traditional sequence labeling tasks are based on the results of the actual label and predicted label of each word. Similar to the conventional classification task, the evaluation indicators of the model effect are precision and recall. During the evaluation of sequence labeling tasks, the definitions of positive and negative examples for classification are different from general tasks. Similarly, in the relationship classification task, two entities can also be divided into four categories according to whether there is a relationship between the actual relationship and the predicted result. This classification is the same as the classification principle of entity recognition. Through the above indicators, the accuracy rate, recall rate, and comprehensive evaluation indicators can be obtained.

In this paper, the accuracy rate *P*, recall rate *R*, and *F*1 values are used to evaluate the results. Different from traditional methods, our method can extract triples without knowing the entity type information. The evaluation indicators are shown in Table 2.

In this paper, the precision rate *P*, recall rate *R*, and *F*1 values are used to evaluate the results. Different from the traditional method, the method in this paper can extract triples without knowing the entity type information. In other words, this paper does not use the labels of entity types to train the model, so entity types do not need to be considered in the evaluation. A triple is considered correct when both the relation type and the position offsets of the two corresponding entities are correct. This paper randomly selects 10% of the data from the test set to create the validation set. In order to ensure the accuracy of the experimental results, this paper will run each experiment 30 times, and then record the average results.

## 5. Results and Analysis

### 5.1. Parameter Settings of the Model

The text entity relationship joint extraction model proposed in this paper is mainly composed of a word vector model and a multi-head attention neural network model. The specific parameters of the training of the two models are shown in Table 3.

**Figure 6:**
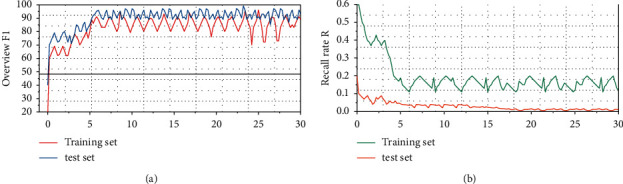
Model training results and loss value curve.

After the model is set with the above parameters, the training result and loss value change curve of the model are shown in Figure 6.

It can be seen from Figure 6(a) that as the number of iterations increases, the training process tends to be stable, and the comprehensive index *F*1 and loss value of the model tend to converge. When the number of iterations reaches 30, the training set *F*1 = 97.88%, and the validation set *F*1 = 95.91%.

It can be seen from Figure 6(b) that due to the two methods of random deactivation rate and *L*2 regularization used in the network, when the iteration exceeds 10 times, the loss value tends to be stable, and the training data *F*1 curve and the validation data *F*1 curve are rising too. At a certain height, it tends to rise gradually, and there is no large fluctuation or decline trend, which shows that the model has excellent effect on the training data, and at the same time, it still has a good performance on the verification data.

### 5.2. Research on the Value of Hyper Parameters for Multi-Head Attention

**Figure 7:**
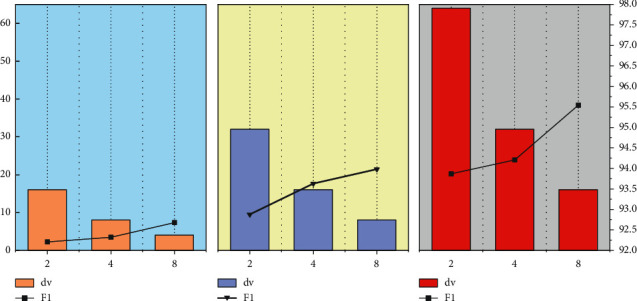
Multi-head attention test data graph.

It can be known from the operation process of the multi-head attention mechanism that its two most important parameters are the number of heads *h*, and the dimension dv of a single head (the size of the attention). In order to study the influence of the multi-head attention hyper parameters on the classification effect of the model, the word vector dimension *dv*, the number of heads *h*, and the attention size were studied, respectively [21]. Under the multi-head attention mechanism, the dimension of the word vector is generally an integer power of 2. In order to facilitate the research, the word vector dimensions are 32, 64, and 128 for experiments, and the number of heads is set to 2, 4, and 8, and the parameters are adjusted to the best state. When the models are all convergent, the evaluation results used are the test set. The experimental results are shown in Figure 7.

As shown in Figure 7, when the dimensions of the three word vector dimensions are the same, with the increase of the number of multi-head attention heads *h*, the classification effect between the maximum number of heads and the minimum number of heads set in the experiment is 0.47%–1.67% %. The more heads *h*, the better the classification effect of the model, which shows the characteristics of the multi-head attention mechanism. By increasing the number of heads *h*, the model can mine the internal information of the text sequence from multiple sub-semantic spaces, and then extract the text. Semantic features to achieve the purpose of improving model performance. When *h* is fixed, the change of the attention size dv affects the classification performance of the model, and the *F*1 of the reduced model also decreases. Taking *h* = 8 as an example, the difference in *F*1 value between the largest *dv* and the smallest *dv* reaches 2.86%. This is because dv is a very important factor affecting the weight coefficient vector. The size of *dv* determines the amount of feature information contained in the vector. When the value is too small, it is easy to cause the loss of the feature information of the text, and it also increases the model's complexity. The complexity of the model further affects the fitting ability of the model and reduces the classification effect of the model. Through comparative experiments, it can finally be confirmed that the optimal hyper parameters of the multi-head attention layer of this model are *h* = 8, *dv* = 16, and the classification effect of the model is the best at this time.

### 5.3. Research on Model Generalization Ability

**Figure 8:**
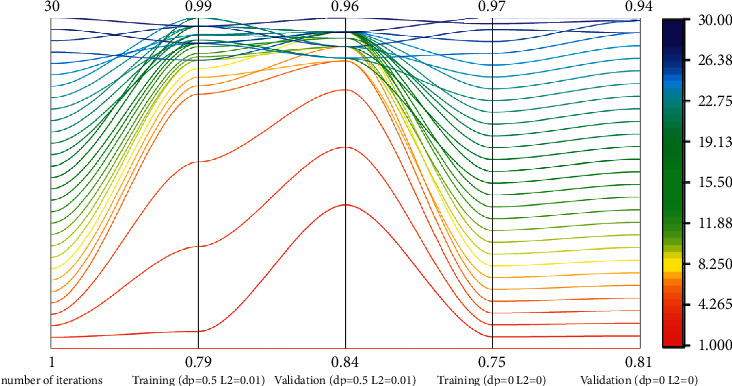
Model generalization capability data map.

The neural network model has strong feature extraction ability, and the model also has a large risk of over-fitting [22]. The generalization ability of the model can be guaranteed and over-fitting can be suppressed by adding random inactivation rate to the model and using *L*2 regularization. In order to study the effect of random inactivation rate and *L*2 regularization coefficient on the classification effect of the model, the model will set the corresponding random inactivation rate and *L*2 regularization coefficient, and the comparison model will not set relevant parameters and layers. The experimental results are shown in Figure 8.

It can be seen from the figure that when the random deactivation method and the *L*2 regularization term are not used, as the number of iterations increases, the curve of the validation set first rises and reaches stability. When the number of training times = 26, with the increase of the number of times, there is an obvious downward trend, but the training set curve has not stopped rising, indicating that the model is over-fitting, and the network complexity at this time is very large; set the dropout layer to DP = 0.5 and use the *L*2 regularization term, and set the coefficient to 0.01 to weaken the network complexity. As the number of iterations increases, the validation set curve and the training set curve rise to 96% and 98%, respectively, and then tends to a gentle upward trend.

On the whole, the training set curve and the validation set curve of the random inactivation method and the *L*2 regularization term model are correspondingly above the training set curve and the validation set curve of the random inactivation method and the *L*2 regularization term model. It is because the neural network using the dropout layer will temporarily disconnect a certain proportion of neurons. Due to the different disconnected neurons, each batch trains a different network. When DP = 0.5, the randomly generated network structure is the most, which can adapt to different data. So the classification effect is the best. *L*2 regularization usually makes the weights as small as possible, and constructs a model with all parameters relatively small. Generally speaking, models with small parameter values are relatively simple, can adapt to different data sets, and have strong anti-disturbance ability, so the curve fluctuation is small and the model performance is good.

The results show that the model with dropout layer added and *L*2 regularization term is better than the model without dropout layer and *L*2 regularization term in classification.

### 5.4. Feature Extractor Selection

The purpose of this experiment is to verify the effectiveness of the feature extraction layer and to find a suitable feature extractor structure. The experimental method is to keep the structure of other layers of the model unchanged, and only change the structure of the feature extraction layer to evaluate the impact of different feature extractors on the model sentence similarity calculation index. The model without feature extraction layer serves as the baseline for the experiments. The structure of the feature extractor participating in the selection experiment is shown in Table 4.

**Figure 9:**
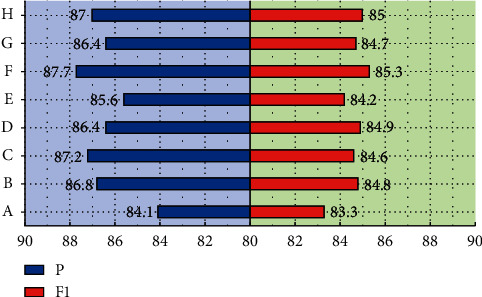
Feature extractor selection experimental results.

In order to facilitate the comparison with other research results, the common evaluation indicators on the corresponding data sets are selected, the accuracy and *F*1 value are used in the MRPC data set, and the Pearson correlation coefficient and the Spear man correlation coefficient are used in the STSB data set. The experimental results of feature extractor selection on the MRPC data set are shown in Figure 9.

As shown in Figure 9, the model with feature extraction layer has advantages in accuracy and *F*1 value indicators, far superior to the model without feature extraction layer. On the accuracy index, *F* performed the best with 87.7%, followed by *C* with 87.2% accuracy. In the *F*1 value indicator, *F* reached 85.3%, which also surpassed other comparison models. The experimental results on the STSB data set are basically consistent with the experimental results on the MRPC data set. The model without feature extraction layer performs the worst, and the best feature extraction layer structure is *F*. The model with feature extraction layer is significantly better than the model without feature extraction layer, which shows the effectiveness of the feature extraction layer, and also shows the effectiveness of the “encode-match-extract” mode. In this paper, Xception is used to extract text features, which enhances the ability of feature extraction and achieves good results in model application.

### 5.5. Model Training and Testing Time

**Figure 10:**
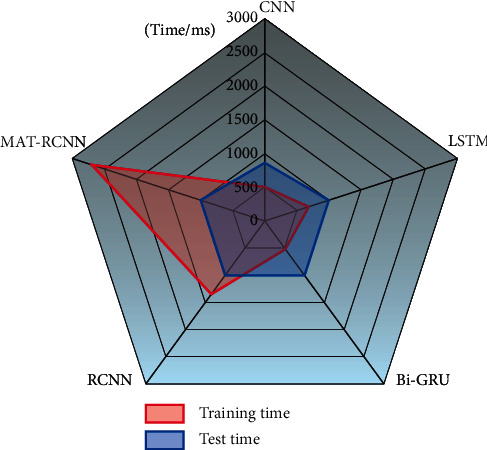
Time consumed data graph of the model.

The training time and test time of each model are studied, and the classification results of the test set are recorded. The training time is the time required for model training, and the test time is the time required to classify the test set. Since the size of the word vector dimension will affect the time consumed, the larger the word vector, the longer the time consumed. When the word vector dimension changes between 50 and 150, the comprehensive index *F*1 and the accuracy rate *P* of the model change by no more than 0.6%. Therefore, in order to objectively test the training time and testing time of each model, the input word vector dimension is set to 128, and the number of iterations is 30, and each model is tested separately. The time consumption of the model is shown in Figure 10.

As shown in Figure 10, the model time-consuming ranking is: CNN < BI-GRU < BiLSTM < RCNN < MAT-RCNN; this is because with the increase of model complexity, the training time and testing time of the model will also increase. Since the CNN model uses different convolution kernels to operate in parallel, the training time and testing time are the least. The training time of the Bi-GRU model is less than that of the Bi-LSTM model, because the two-gate design of the GRU model is simpler and less time-consuming than the three-gate design of the LSTM. Because the model structure of this model is more complicated than that of the comparison model, the training time is longer, but the test time is not much different from that of the comparison model. In practical applications, only the trained model needs to be loaded to calculate the test data. The time required for classification corresponds to the test time. The model in this paper can complete the classification of all the data in the test set in 1005 ms, which cannot be achieved manually.

## 6. Conclusion

To sum up, this paper proposes a joint extraction model of text entity relationship based on multi-head attention neural network based on the research of previous entity relationship extraction algorithm and language representation model. It is an entity-relationship joint extraction model based on feature enhancement, and a three-layer linear architecture model of text feature extraction layer, main entity recognition module, and object entity-relation recognition module is designed. In terms of enhancing the relevance of the text domain, this paper is preinstalled on the larger Baidu Baikal data set to make the training model more relevant to the relation extraction task. In terms of feature enhancement, entity features and part-of-speech tagging features are added, so that the model can obtain more accurate entity relationship information. Extraction in the relationship between the host entity and the object entity modules add a multi-head attention mechanism and an improved recurrent neural network, respectively, which enhances the learning ability of sequential features between tokens and further improves the effect of the model in this paper. Finally, through experimental comparison, the accuracy rate of the model in this paper reaches 87.7%, and the entity relationship can be extracted at the fastest at 1005 ms. In terms of text correlation, feature extraction effect, main entity relationship extraction effect, etc., the model in this paper proves that it has certain advantages in the task of information extraction, which effectively improves the efficiency of information extraction and has a strong practical value. The joint extraction model of text entity relationship in this paper only extracts the relationship of a pair of entities and cannot be applied to the extraction of multiple pairs of entities in a single sentence. In the follow-up research, we need to find more advanced algorithms to achieve the extraction of many-to-entity and many-to-relationships.

## Figures and Tables

**Table 1:** Server configuration.

CPU	RAM (GB)	GPU	Survive (GB)	Hard disk
Intel(R) XEON(R) CPU E5-2683 v3 @ 2.00 GHz (28 cores, 56 threads)	62	NVIDIA GEFORCE GTX1080Ti	12	3.1 TB SSD

**Table 2:** Evaluation metrics.

Evaluation indicator	Indicator content

Accuracy	*P* = Correctly extracting the number of entities/quantity of extracted entities
Recall rate	*R* = Correctly extracting the number of entities/total number of entities in data concentration
Overview	*F*1=2*∗P∗R*/(*P*+*R*)

**Table 3:** Word vector training parameter.

Parameter description	Word vector dimension	Word frequency threshold	Random number generator

Set value	128	0	1
Parameter description	Window size	Random down sampling configuration	Learning rate
Set value	5	0.001	0.025

**Table 4:** Feature extractor structure.

Structure name	None	InceptionV3	DenseNet121	DenseNet169

Code	A	B	C	D
Structure name	DenseNet201	Xception	InceptionResnetV2	ResNet50
Code	E	F	G	H

## Data Availability

The data used to support the findings of this study are available from the corresponding author upon request.
